# Microbial hydrogen consumption leads to a significant pH increase under high-saline-conditions: implications for hydrogen storage in salt caverns

**DOI:** 10.1038/s41598-023-37630-y

**Published:** 2023-06-29

**Authors:** Nicole Dopffel, Kyle Mayers, Abduljelil Kedir, Edin Alagic, Biwen Annie An-Stepec, Ketil Djurhuus, Daniel Boldt, Janiche Beeder, Silvan Hoth

**Affiliations:** 1 NORCE Norwegian Research Center AS, Nygårdsgaten 112, 5008 Bergen, Norway; 2 Equinor ASA, Stavanger, Norway

**Keywords:** Environmental sciences, Biogeochemistry, Environmental microbiology

## Abstract

Salt caverns have been successfully used for natural gas storage globally since the 1940s and are now under consideration for hydrogen (H_2_) storage, which is needed in large quantities to decarbonize the economy to finally reach a net zero by 2050. Salt caverns are not sterile and H_2_ is a ubiquitous electron donor for microorganisms. This could entail that the injected H_2_ will be microbially consumed, leading to a volumetric loss and potential production of toxic H_2_S. However, the extent and rates of this microbial H_2_ consumption under high-saline cavern conditions are not yet understood. To investigate microbial consumption rates, we cultured the halophilic sulphate-reducing bacteria *Desulfohalobium retbaense* and the halophilic methanogen *Methanocalculus halotolerans* under different H_2_ partial pressures. Both strains consumed H_2_, but consumption rates slowed down significantly over time. The activity loss correlated with a significant pH increase (up to pH 9) in the media due to intense proton- and bicarbonate consumption. In the case of sulphate reduction, this pH increase led to dissolution of all produced H_2_S in the liquid phase. We compared these observations to a brine retrieved from a salt cavern located in Northern Germany, which was then incubated with 100% H_2_ over several months. We again observed a H_2_ loss (up to 12%) with a concurrent increase in pH of up to 8.5 especially when additional nutrients were added to the brine. Our results clearly show that sulphate-reducing microbes present in salt caverns consume H_2_, which will be accompanied by a significant pH increase, resulting in reduced activity over time. This potentially self-limiting process of pH increase during sulphate-reduction will be advantageous for H_2_ storage in low-buffering environments like salt caverns.

## Introduction

Decarbonisation of industrial and societal activities is anticipated to hinge on “Power-to-Gas” technologies, which use electricity derived from solar or wind sources to produce hydrogen (H_2_), a universal energy carrier. H_2_ can be utilized, transported or stored for later use for several industrial sectors like chemical industry, heavy transport and steel production, “Gas-to-Power”. Storage of H_2_ is needed to buffer daily to seasonal variations in energy supply and demand^1–3^. Underground storage in salt caverns is suggested to be the ideal option for large-volume storage of H_2_ when the gas can be injected into a cavern and can be withdrawn flexibly for energy generation^4–6^. This process is very similar to the current storage of natural gas or oil. Salt caverns are solution mined large underground cavities inside a salt layer or salt dome by gradually dissolving the salt with freshwater or seawater. The resultant voids are several tens of meters in diameter and several hundreds of meters in length, with geometric cavern volumes of up to 10^6^ cubic meters and maximum pressures of up to 300 bar. They are proposed to be ideal for short- to mid-term storage, with opportunities for rapid injection and withdrawal of gases for energy balancing. Major benefits of salt cavern storage are the available high volumes and relatively low operational costs^5^. As the demand for more storage sites increases, and upfront capital investments for salt caverns are high (~ 30 M€/onshore cavern^7^) there is an increasing need for improved understanding of possible microbiologically triggered subsurface reactions of H_2_ within the salt cavern. These reactions can potentially reduce the caloric value as well as leading to health/safety/environmental relevant generation of H_2_S, which constrain the operational window and requires purification measures. Currently there are only a hand full of salt cavern sites used for H_2_ storage worldwide^5^, without reported failures but operational data is not available.

Salt caverns are, like most subsurface environments, not sterile but harbour diverse microbial organisms^8–10^. Specially adapted extremophiles (halotolerant or halophilic) can live in or even require high-salt conditions for their survival^11,12^. Although high osmotic stress is suggested to cause energetic constraints^13^ by forcing the microorganisms to spend high amounts of energy for osmoregulation, i.e., production of compatible solutes; a higher salinity in salt cavern brines does not necessarily prevent the risks of microbial presence and/or activity. In case of H_2_ storage, the microorganisms will be in direct contact with the stored H_2_ for an extended period (up to several months for mid-/long-term storage). H_2_, being an excellent and ubiquitous electron donor, is an important driver for microbial activity in living environments^14,15^, which is in stark contrast to natural gas (CH_4_). Microbial activity in H_2_ filled salt caverns could induce a variety of processes and risks related to them: gas volumetric effects, gas composition changes and purity loss by e.g. sulphate-reducing microbes (SRM) forming the toxic gas H_2_S (see reaction 1), methanogenic archaea forming CH_4_ (see reaction 2), overall causing a reduced energetic value^16,17^.

1 $${1}/{\text{4SO}}_{{4}}^{{{2} - }} + {\text{H}}_{{2}} + {1}/{\text{4H}}^{ + } \to {1}/{\text{4HS}}^{ - } + {\text{H}}_{{2}} {\text{O}}$$

2 $${1}/{\text{4HCO}}_{{3}}^{ - } + {\text{H}}_{{2}} + {1}/{\text{4H}}^{ + } \to {1}/{\text{4CH}}_{{4}} + {3}/{\text{4H}}_{{2}} {\text{O}}$$

In field trials for H_2_ storage in porous reservoirs and aquifers, microbial H_2_ consumption and conversion into CH_4_ has been described several times^18,19^. However, for salt caverns the overall microbial risks are not well studied and for cavern operators the extent of the microbial problem is unclear. For example, it is not known whether or how microbial H_2_ oxidation processes will occur in salt caverns and if so, at which speed and how pronounced. Some modelling approaches have indicated potential H_2_S formation^20,21^. However, these models are based on kinetic rates of sulphate reducers grown under standard laboratory conditions, and it can be assumed that a) growth and consumption is different with H_2_ as an electron donor, and b) that extremely halophilic strains show different rates due to their energy expenditures on osmoregulation. For better prediction of the microbial risks and the underlying economic risks, it is therefore necessary to study specific halophilic microbial rates to estimate microbial H_2_ oxidation under high-salt conditions.

Therefore, the aim of this study was to investigate known halophilic H_2_-consuming microbial strains to obtain not only H_2_ consumption rates but also to find some key parameters that influence and/or can be used as indicators for microbial H_2_ consumption. Furthermore, we compared our findings with microbial enrichments from a brine sampled in a salt cavern located in Northern Germany. These enrichments were incubated with H_2_ for over 150 days at the cavern specific temperature range. Our study clearly shows that microbial H_2_ consumption is a relevant topic for salt cavern storage and delivers important kinetic data on H_2_ consumption of both cultured and environmental samples.

## Material and methods

### Source of organisms

The two halophilic cultures *Desulfohalobium retbaense* DSM5692^22^ and *Methanocalculus halotolerans* DSM14092^23^ were purchased from the DSMZ (German Collection of Microorganisms and Cell Cultures GmbH). The original cavern brine was sampled at a cavern field located in Northern Germany. Samples were taken at the wellhead. Before taking samples the first brine was discarded (around of 10 min flushing) to avoid sampling the brine standing in the well. Afterwards the samples were filled into sterile and anoxic glass bottles under continuous nitrogen flush to preserve anoxic conditions and then immediately shipped to the lab. Brine properties are: salinity 27% (wt/wt), pH 7.4, sulphate 4190 + / − 57 mg/L, total inorganic carbon 84.9 + / − 0.4, total organic carbon 7.61 + / − 1.1 mg/L.

### Standard culture conditions

Both reference strains were routinely cultured in their specific media given by the DSMZ. For *D. retbaense* DSM5692: 1 g/L NH_4_Cl, 0.3 g/L K_2_HPO_4_, 0.3 g/L KH_2_PO_4_, 20 g/L MgCl_2_ × 6 H_2_O, 100 g/L NaCl, 2.7 g/L CaCl_2_, 4 g/L KCl, 3 g/L Na_2_SO_4_, 1 mL/L trace element solution SL-10, 0.3 g/L Na_2_S × 9 H_2_O—pH 7.2. For *M. halotolerans* DSM14092: 1 g/L NH_4_Cl, 0.3 g/L K_2_HPO_4_, 0.3 g/L KH_2_PO_4_, 3.2 g/L MgCl_2_ × 6 H_2_O, 50 g/L NaCl, 0.6 g/L CaCl_2_, 0.17 g/L KCl, 3 g/L Na_2_SO_4_, 10 mL/L modified Wolins mineral solution, 0.3 g/L Na_2_S × 9 H_2_O, 2 g/L NaHCO_3_—pH 7.2. Carbon sources and yeast extract were added separately to the bottles depending on the experiment. Both microorganisms were incubated at their respective optimal temperature of 37 °C. Standard growth for *D. retbaense* was on 24 mM lactate with 0.1% yeast extract and 0.1% peptone. *M. halotolerans* was routinely cultivated with 20 mM acetate, 20 mM formate, 0.05% yeast extract and 80% H_2_, 20% CO_2_. Growth was confirmed by gas production (H_2_S or CH_4_).

### H_2_ consumption experiments

Bottles (total volume 58.35 mL) were always filled with 25 mL medium. For growth on H_2_, *D. retbaense* cultures were amended with 24 mM acetate and 0.35 mL modified Wolins vitamin solution, inoculum 10% (2.5 mL) of a culture grown on lactate for 7 days. The incubations of *M. halotolerans* were amended with 20 mM acetate, 35 mM formate, 0.05% yeast extract, inoculum 10% (2.5 mL) of a culture grown on 80%/20% H_2_/CO_2_ for 4 days. Different amounts of H_2_ were added to headspace to obtain 10% (~ 4 mL), 40% (~ 11 mL) and 100% (~ 25 mL)(rest gas: N_2_) of the total headspace volume. Fluid and gas volume was always kept constant or defined volumes were withdrawn and considered in the calculations. Because *M. halotolerans* requires CO_2_ for methanogenesis, 5% of the gas phase was CO_2_, which was re-supplied after gas analysis at each sampling point. Incubation temperature was 37 °C for both strains. Each experiment was conducted twice independently. Sterile controls contained the media and all additions but were not inoculated. The original cavern brine was anoxically filled in sterile bottles and the headspace was flushed with 100% H_2_. Several different enrichments were set up either containing only 100% H_2,_ , 20 mM acetate plus 0.04% yeast extract plus 100% H_2_ or 10% CO_2_ + 90% H_2_. All experimental conditions can be found in the supplement Table S1. Incubation temperatures was 30 °C. All bottles were stored upside down during incubation to minimize loss of H_2_ due to diffusion through the rubber stopper. Still, we observed diffusion through or reaction with the stoppers especially over longer incubation periods.

**Table 1:** Measured and calculated results for hydrogen consumption and growth of *Desulfohalobium retbaense* (incubation at 37 °C for 71 days, starting pH 7.5, initial cell number 2.80E+10 cells/mL), *Methanocalculus halotolerans* (incubation 37 °C for 10 days, starting pH 7.4, initial cell number 7.2E+09 cells/mL) and an original salt brine sample (incubated at 30 °C, starting pH 7.4).

	Volume of H2 in headspace at day 0 (%)	Volume H_2_ in headspace day 0 (mL)	Hydrogen consumed/lost (mL)	Acetate consumed (mM)	Cell number at end of incubation (cells/mL)	pH at end of incubation
*D. retbaense*	10	3.9 ± 0.1	3.9 ± 0.0	0 ± 0.6	4.10E+10	8.9 ± 0
	10	3.5 ± 0.1	1.8 ± 0.0			
*D. retbaense*	40	11.5 ± 0.7	7.5 ± 0.9	0.7 ± 0.1	4.13E+10	9.2 ± 0.1
*D. retbaense*	100	24.9 ± 0.1	14.3 ± 2.4	1.1 ± 3.7	4.12E+10	9.1 ± 0
Sterile medium	10	3.4 ± 0.2	0	–	–	7.3 ± 0.1
Sterile medium	40	11.3 ± 0.2	1 ± 0.2	–	–	7 ± 0
Sterile medium	100	24.5 ± 0.4	1.5 ± 0.2	–	–	6.9 ± 0.1
*M. halotolerans*	10	3.4 ± 0.2	3.4 ± 0.6	0.2 ± 0	4.04E+10	8.6 ± 0.1
	10	4.1 ± 0.5	4.1 ± 0.5			
*M. halotolerans*	40	11.9 ± 0.9	11.9 ± 0.9	0.5 ± 0.1	4.45E+10	8.5 ± 0.1
*M. halotolerans*	90	23.6 ± 1.4	22.8 ± 2.2	2.2 ± 0.6	5.08E+10	8.8 ± 0.1
Sterile medium	10	3.6 ± 0.3	0.4 ± 0.1	–	–	7.4
Sterile medium	40	10.5 ± 0.3	0.1 ± 0.1	–	–	7.8
Sterile medium	90	22.5 ± 0.2	2.3 ± 0.3	–	–	7.7
Salt cavern brine	100	54.1 ± 1.3	2.2 ± 0.9	–	n.d	7.8 ± 0.1
Salt cavern brine + acetate/yeast extract	100	57.3 ± 0.4	5.8 ± 0.1	0.9 ± 0.3	n.d	8.5 ± 0.1
Sterile water	100	56.2	1.6	–	–	–

All experiments were performed in 58 mL glass bottles containing 25 mL medium/brine.

*n.d.* not determined.

### Sampling and calculations

Pressure measurements, gas analysis and liquid sampling were performed in regular intervals during the incubation: For *D. retbaense* every week, for *M. halotolerans* every day/every second day and for the original brine enrichment every 50 days. Pressure measurements of the experiments were conducted before and after each sampling session. When the pressure fell below 25 mbar (detection limit of the microGC), the bottles were re-pressurized with N_2_ (for *D. retbaense)* or N_2_/CO_2_ (for *M. halotolerans)*. At some sampling points 1 mL liquid were withdrawn for pH, HPLC and cell number determination. To calculate the amount of H_2_ in the bottles, the ideal gas law (3) was used to correlate temperature (T in Kelvin), pressure (p) in the bottles with atmospheric pressure—water vapor pressure at the given temperature (in Pascal) and the gas constant 8.3144 (J/mol K). The measured composition of the gas phase (%) and the known volume of the bottle (58.3 mL—liquid medium + additions) was used to calculate gas volume of H_2_ the bottles containing the medium V (in m^3^).

3 $${\text{p}} \times {\text{V}} = {\text{n}} \times {\text{R}} \times {\text{T}}$$

The resulting mol of H_2_ was calculated into mL by assuming STP (Standard temperature and pressure) (1 mol = 22.4 L). For each sample we calculated the volume of H_2_ before and after the sampling procedure by measuring headspace pressure at the beginning and at the end of the sampling. The loss of H_2_ in between is related to withdrawal of gaseous and liquid samples. This loss through sampling was calculated and subtracted from the calculated first pressure value to obtain the volume of consumed H_2_. Absolute maximum rates were obtained by calculating the slope of H_2_ loss during days. Relative rates were obtained by normalizing the H_2_ values to the initial starting value, resulting in %-loss over time.

### Analytical methods

Gas composition was measured with a micro gas chromatography (microGC) 490 (Agilent) by directly measuring the gas in the headspace of the serum bottles. Pressure in the bottles was measured using a pressure sensor from Sensortechnics 0-3 barg Press D/C 2916 with an individual set-up for direct measurement of the headspace of serum bottles. Liquid samples were analyzed by using liquid chromatography of Agilent 1260II UHPLC equipped with a Flexible pump, autosampler, 1260 RI, and 1260 DA HS detectors. All analytes were identified and quantified based on their respective reference standard calibration curves. For determining cell numbers, we isolated DNA from 1 mL of sample which was withdrawn using syringes and centrifuged for 20 min at 13,000 rpm. The pellet was frozen for several hours at − 80 °C and after that at − 20 °C. This procedure was necessary because the pellets did not freeze at − 20 °C due to the high salt content. Prior to DNA isolation, the pellet was ultrasonicated for 5 min, frozen for several hours at − 80 °C, and again ultrasonicated 5 min. DNA was isolated using the Blood&Tissue Kit (Qiagen) following manufacturer’s instructions. Copy numbers were measured via digital droplet PCR (ddPCR; BioRad) using Dsr1 primer for sulphate-reducing bacteria^24^ or standard 16S rRNA Archaea^25^. The ddPCR reactions were run with a total volume of 20 µL on a DX200 instrument (BioRad) using 1 × EvaGreen supermix (BioRad) and 250 nM (final concentration) of primers. Complete PCR reactions were emulsified with QX200 Droplet Generation Oil for EvaGreen using the QX200 Droplet Generator and then transferred to a 96-well plate. PCR reactions were performed in a C1000 Touch Thermocycler with deep-well module (BioRad) using the following program: 95 °C for 15 min, 40 cycles of 95 °C for 30 s, 57.1 °C (sulphate-reducing bacteria) or 63.1 °C (archaea) for 1 min, 4 °C for 5 min, 90 °C for 10 min and finally an infinite hold at 4 °C. Plates were equilibrated to room temperature for at least 10 min before being analysed on a QX200 Droplet Reader (BioRad). Thresholds for positive and negative droplets were manually set using positive (Bacteria or Archaea cultures) and negative (ultra-pure water) controls. The copy numbers were calculated to cell numbers based on the available genome of *D.retbaense* with 1 gene copy of dsr1/cell^26^. The genome of *M. halotolerans* is not available and therefore we assume 1.7 gene copy numbers/cell of 16S rRNA in general Archaea based on the rrnDB database (value taken on the 24.10.2022).

## Results

### Hydrogen consumption during halophilic sulphate reduction

**Figure 1:**
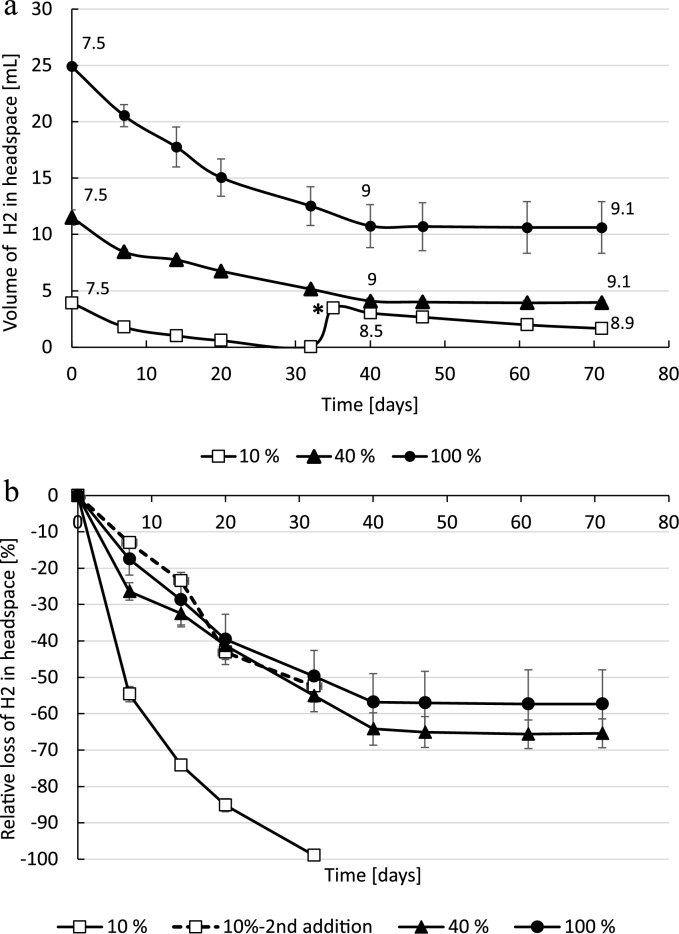
(**a**) Consumed hydrogen of *Desulfohalobium retbaense* in ml over time measured in the headspace of bottles incubated at near atmospheric pressure and 37 °C. Bottles were amended with 100% H_2_ (filled circle, solid line), 40% H_2_ (filled triangle, solid line) and 10% H_2_ (open square, solid line). The star * at 35 days indicates re-feeding the bottles with 10% H_2_. pH measured at the day are given above the line. (**b**) Values plotted in loss in % over time. Error bars indicate deviation from the mean of duplicates. At day 35, 10% of H_2_ was again added to the 10% H_2_ bottles (dashed line—2nd addition).

The halophilic sulphate-reducing bacteria *Desulfohalobium retbaense* consumed H_2_ in the headspace over several weeks. In the 10% H_2_ bottles the cells consumed all available H_2_ (around 4 mL H_2_) after 20 days while the incubations with 40% H_2_ (initial 11 mL H_2_) and 100% H_2_ (initial 25 mL H_2_) H_2_-headspace were only partially consumed (Fig. 1A). We observed maximum rates of 0.31, 0.44 and 0.62 mL/day for 10, 40 and 100% H_2_ respectively. In the set-ups with 10% H_2_, we added 10% H_2_ at day 35 after the first consumption but subsequently consumption rate was significantly slower with a maximum rate of 0.09 mL/day. In total *D. retbaense* consumed 3.8 + 1.8 (initial 10% H_2_ + renewed addition of 10% H_2_), 7.5 (40% H_2_ set-up) and 14.3 (100% H_2_ set-up) mL of H_2_, which correspond to the relative amounts of 99% + 52%, 65% and 57% H_2_ (Fig. 1B). The experiment was repeated twice with a standard deviation variation of the maximum rates between 7 and 23% (see all data in suppl. Table 2). In sterile controls 0 mL for 10% H_2_ , 0.9 mL for 40% H_2_ and 1.5 mL for 100% H_2_ were lost through diffusion out of the rubber stopper or by other chemical reactions, which shows that some loss occurs abiotically. During the incubation, either no or only minor amounts of acetate were consumed (0 mM with 10% H_2_, 0.7 mM with 40% H_2_ and 1.1 mM with 100% H_2_) (Table 1), which shows that acetate was not limiting. From an initial 2.80E+10 cells/mL, cell numbers did only slightly increase with all H_2_ concentrations to around 4E+10 cells/mL indicating that the strain was not able to build up substantially more cell mass.

Only minor amounts of H_2_S were detected in the headspace with a maximum value of around 4000 ppm at day 32 in one duplicate of the 40% H_2_ bottles. However, at the next sampling stage the H_2_S concentration was again back to the background level (suppl. Fig. S1). This indicates a changing H_2_S/HS^−^ dissolution in the liquid, which was confirmed by pH measurements, showing a significant increase in pH over time. Starting from pH 7.5 at day 0 and resulting in a final pH of 8.9 for 10% H_2_ and pH 9.1–9.2 for the 40% H_2_ and 100% H_2_ bottles (Fig. 1A; Table 1, all pH values over time can be found in supplemental Table 3). Sulphate was not quantified but was in excess (21 mM) in the media allowing for theoretical consumption of 47 mL H_2_. We therefore assume that sulphate was never limiting.

### Hydrogen consumption during methanogenesis

**Figure 2:**
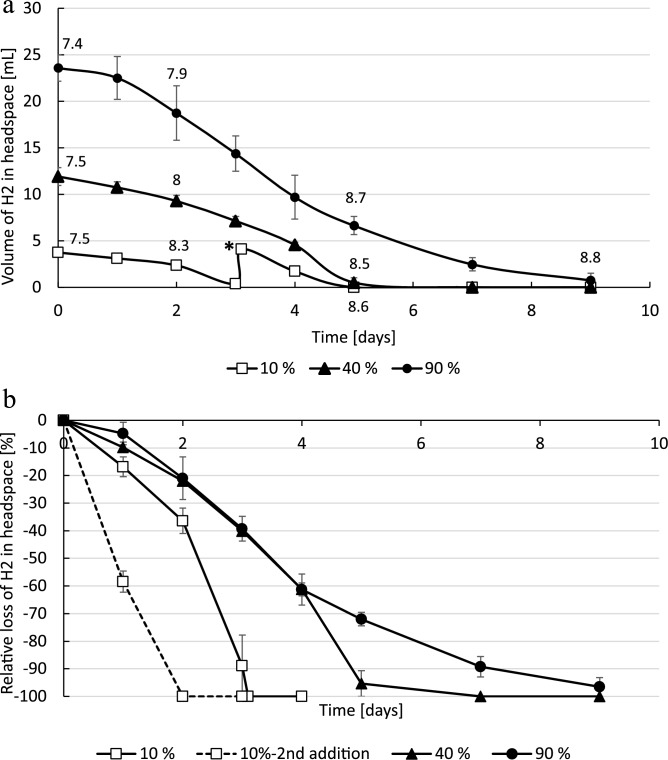
(**a**) Consumed hydrogen of *Methanocalculus halotolerans* in mL over time measurable in the headspace of bottles incubated at near atmospheric pressure and 37 °C. Bottles were amended with 90% H_2_ (filled circle, solid line), 40% H_2_ (filled triangle, solid line) and 10% H_2_ (open square , solid line). The star * at 35 days indicates re-feeding the bottles with 10% H_2_. pH measured at the day are given above the lines (10% H_2_ day 5 is under line). (**b**) Values plotted in loss in % over time. At day 3, 10% H_2_ was re-added in the 10% set-ups (dashed line—2nd addition). Error bars indicate deviation from the mean of duplicates.

The halophilic methanogen *Methanocalculus halotolerans* consumed H_2_ much faster compared to *D. retbaense* but still slow compared to other reported methanogenic growth^27,28^. In all set-ups all H_2_ was consumed (Fig. 2A) with maximum rates of 1.1, 4.1 and 4.7 mL/day for the 10% H_2_, 40% H_2_ and 90% H_2_ set-ups. Re-addition of 10% H_2_ in the 10% H_2_ bottles led to an increased activity with a consumption rate of 1.7 mL/day (Fig. 2B). The experiment was conducted twice with a standard deviation between maximum rates between 15 and 37%. CH_4_ was produced accordingly in all set-ups with consumption of CO_2_ (suppl. Fig. S2). CO_2_ was always re-added when values dropped below 1.5%, to not limit growth. Formate was completely consumed after 1 day in all set-ups also the non-hydrogen controls. Acetate was consumed only in low concentrations for the 10% H_2_ (0.2 mM acetate consumed) and 40% H_2_ bottles (0.5 mM acetate consumed). 2.2 mM acetate were consumed in the 90% H_2_ bottles (Table 1). Cells numbers increased from 7.2E+09 to 4E+10 cells/mL for the 10% H_2_, to 4.5E+10 cells/mL for the 40% H_2_ and 5.1E+10 cells/mL and 90% H_2_ set-ups. Similar to *D. retbaense*, pH increased in all the *M. calculus* cultures. Starting from a pH of 7.2, it increased to pH 8.6, 8.5 and 8.8 (Table 1; Fig. 2A; Supplemental Table 3).

### Hydrogen consumption of a real salt cavern brine community

**Figure 3:**
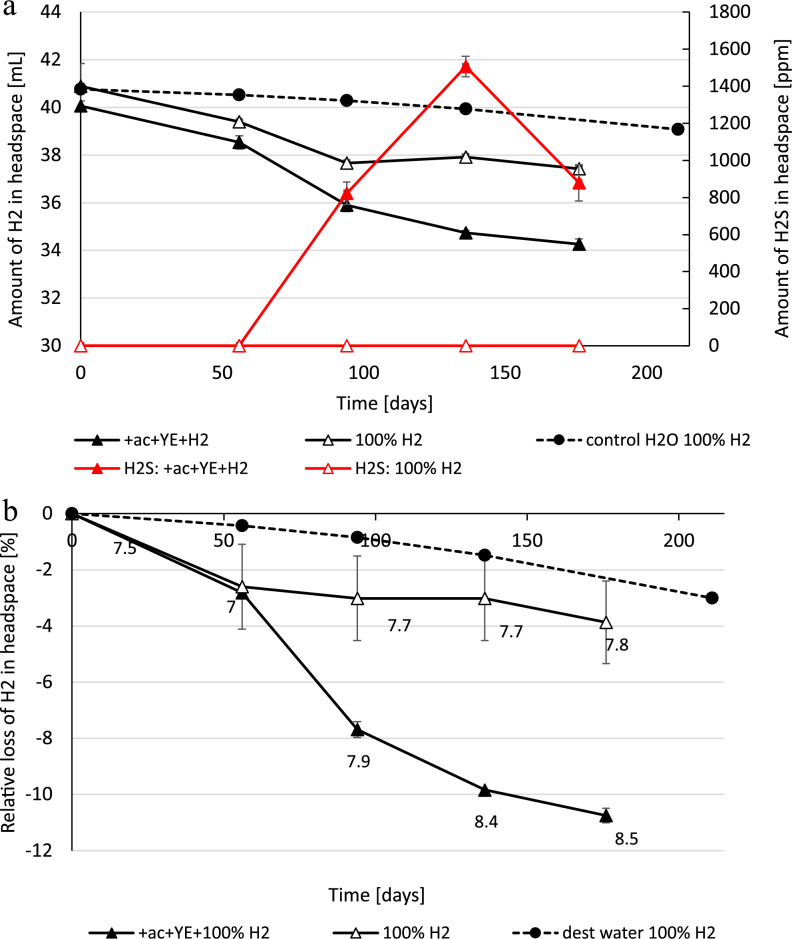
(**a**) Consumed hydrogen of an original salt cavern brine in mL over time measurable in the headspace of bottles incubated at near atmospheric pressure and 30 °C. Bottles were amended with 100% hydrogen (emty triangle, solid line) or with addition of 20 mM acetate and 0.04% yeast extract (solid triangle, solid line). H_2_S measured in ppm in the headspace is given on the secondary axis for the acetate + yeast extract bottles (red line). Diffusion loss in an only sterile lab water bottle is shown with filled circle, dashed line. (**b**) Values plotted in % loss. pH values are given at the single sampling points in the graph. Error bars indicate deviation from the mean of duplicates.

The sampled cavern is located in Northern Germany in the Permian Zechstein Group salt layer. It has been filled with brine since the initial leaching several decades ago. The brine has a salinity of 27%, pH 7.4 and a high sulphate content of 4190 ± 57 mg/L. The pure cavern brine was incubated in serum bottles with a headspace of 100% H_2_. We incubated for 176 days at 30 °C which is relevant for this specific cavern. We added acetate and yeast extract to one set of bottles to trigger microbial activity, the other set was pure brine without any additional supplements. Sterile water with 100% H_2_ lost 1.6 mL of H_2_ during the incubation of 211 days. The brine sample incubated with pure 100% H_2_, consumed 1.7 mL H_2_ on average (Fig. 3A). One bottle showed significantly lower total H_2_ loss compared to the duplicate, although initial consumption rate was similar. In contrast with addition of acetate and yeast, 5.3 mL H_2_ (corresponding to ~ 11%) (Fig. 3B) was consumed in both bottles with the maximum rate of 0.069 mL/day and a total of 0.9 mM acetate was consumed after 136 days. At day 100 no acetate was consumed although sulphate-reduction was already visible. This indicates that the first carbon from either the brine or the yeast extract was used before acetate was consumed. H_2_S was first detected after 94 days with a maximum of 1505 ppm at day 136 in the H_2_ + acetate + yeast bottles and declined afterwards due to the increase in pH. In the pure H_2_ bottles, H_2_S was not detected in the headspace, but black precipitates were visible on the glass wall, which indicates possible FeS formation caused by H_2_S reacting with ferrous iron ions. pH first decreased to around 7 and afterwards increased to 8.5 in the H_2_ + acetate + yeast bottles and to pH 7.8 in the pure H_2_ bottles. Interestingly at the last sampling point (after 176 days), trace amounts of CH_4_ (< 0.1%) were detected in one incubation of the acetate + yeast + H_2_ experiment. CO_2_ concentrations were always below 0.1%. It is possible that the CH_4_ production is attributed to acetogenic or methylotrophic methanogens or the detected trace amounts of CO_2_ are indeed sufficient. Further investigations are needed to confirm this observation.

Cultures containing 10% CO_2_ + 90% H_2_ were established to investigate activities of hydrogenotrophic methanogens and acetogens, but no CH_4_ or acetate production was observed at any time. It must be mentioned that the addition of CO_2_ led to a pH decrease to pH 6.5 because of the lack of buffering compounds in the brine, which might have affected the microbial community.

## Discussion

### Hydrogen consumption of halophilic microbes

With the increased interest in storing H_2_ in the subsurface and especially in salt caverns, it becomes important to understand the risks of microbial H_2_ consumption during storage within high-salt environments. Many Bacteria and Archaea can live and even thrive under high salt concentrations. Although high salinity environments are hostile for many groups of microorganisms^12,29^, diverse microbial communities can still be found in high-saline environments like salt lakes and also salt caverns^30^. However, a distinction between halophilic and halotolerant must be made. Halophiles range from slightly to moderate to extreme require a certain amount of salt to grow and show their optimum growth behavior typically at salinities above 100 g/L (Oren, 2011) and can tolerate up to extreme ranges (> 200 g/L). Halotolerant microbes do not need high salt concentrations but can tolerate it to a certain degree. Salinity is a common stress factor and microorganisms have different strategies to adapt to the osmotic pressure imposed by the ionic strength of the surrounding environment^31^. Both strategies, the “salt-in”- or the “osmotic solutes” strategy, require a certain amount of energy (ATP) to uphold cell activity. Either ATP is used directly in the production of compatible solutes (Oren, 2006), or ATP is required to uphold the correct sodium and potassium gradient between the inside and the outside of the cell. This is also the reason why many halophilic microbes living at the “energetic edge”, using much energy for upholding their cell stability, tend to grow slower compared to non-halophilic counterparts. Therefore, it is very important to specifically assess H_2_ oxidation by halophilic H_2_-oxidizers because the relative kinetic rates in such environments are largely unknown.

To estimate the H_2_ consumption rates and associated effects in high-salt subsurface environments, we investigated and cultured two halophilic microorganism which belong to the two most relevant metabolic groups regarding H_2_ consumption: (i) Sulphate reduction is a very widespread metabolism with many different microbial groups potentially using H_2_ as electron donor in the presence of sulphate as electron acceptor^32^, resulting in the production of the toxic and corrosive gas H_2_S; (ii) Methanogenesis from H_2_ and CO_2_ is another highly relevant H_2_ consumption process, which recently received major attention for potentially producing “green” methane (biomethanation) in the subsurface after injecting renewable H_2_ with captured CO_2_^33^. The two investigated strains in our study are halophilic with *Desulfohalobium retbaense* at a salt optimum at 12% growing up to 24%^22^ and *Methanocalculus halotolerans* with the optimum of 5% growing up to 12.5% salinity^23^. Both strains were originally described to be able to use H_2_ for growth, both requiring acetate as an additional carbon source. To estimate the potential H_2_ consumption kinetics in the high-saline subsurface we carefully cultured the strains with different volumes of H_2_ in the headspace. We clearly observed H_2_ consumption over time with increasing consumption rates with increasing H_2_ concentrations. When comparing the maximum rates, we observe only a doubling of rate (0.6 mL/day) for 100% H_2_ compared to 10% H_2_ (0.3 mL/day). Interestingly, when looking at the H_2_ loss relative to the initial concentration, growing with initial 10% H_2_ instead of 40% or 100% has the highest relative rate (= loss in %). Providing all nutrients in excess and starting with a high cell number, we would have expected a much more pronounced consumption with 100% H_2_. This observation might be due to physiological constrains, like limitations of H_2_ transport to and into the cell. H_2_ is normally a very scarce electron donor in the environment, typically in the nmol range. So high amounts of H_2_ could have a negative effect on cellular H_2_ uptake or involved hydrogenase enzymes but details need to be further investigated. Overall, *D. retbaense* was not able to oxidize all the provided H_2_ in the 40% and 100% H_2_ set-ups although acetate and sulphate was provided in excess. This lack of continued activity or consumption can be explained by an intense pH increase during H_2_-oxidation. It can be seen in the reaction rate of H_2_-dependent sulphate reduction (1) that this reaction is a proton consuming process leading to an increase in surrounding pH^34,35^. This also explains the near complete absence of gaseous H_2_S throughout our study. At higher pH H_2_S will be in its highly soluble form HS^−^ (HS^−^  + H^+^  ⇋ H_2_S). HS^−^ is a weak acid which should partly counteract the pH increase but clearly the intense H_2_ oxidation is the main driver and overrides the HS^−^ effect. In biogas plants and biocorrosion studies it is commonly reported that intense sulphate-reduction can lead to a pH increase^36,37^. As biogas and corrosion involves also intermediate H_2_ release, the observed pH increase might be also a direct effect of microbial H_2_ oxidation. Although it has been long known that H_2_ is an electron donor for SRMs, it was never clearly described that the significant pH effect limits the microbial activity when growing on pure H_2_. As the pH approached pH 9, which is beyond the acceptable limit for *D. retbaense*, its activity ceased. Another inhibiting effect could be significant amounts of dissolved HS^−^ (theoretically based on the amount of H_2_ consumed: 1.4, 1.9 and 3.6 mL for the 2 × 10% H_2_, 40% H_2_ and 100% H_2_ respectively), which might have an additional inhibitory effect on the activity although assume that this inhibition is rather small as sulphate reducers tend to have a high tolerance for HS^−^. When growing on lactate as an electron donor, pH did not rise significantly with an end value of pH 7.8 after 7 days with H_2_S concentrations far beyond 15% of the headspace (suppl. Table 4). We assume that this is due to the production of the organic acid acetate and CO_2_^38^, which both will lead to a decrease in pH. So, the observed pH increase in culture growing on only H_2_ is purely associated to the enzymatic process of H_2_ oxidation.

We were however not able to correlate the overall volume of H_2_ consumed with the pH. In the 40% H_2_ set-up the strains consumed 6.6 mL (consumption minus the diffusion loss observed in the sterile controls) and in the 100% H_2_ set-up it consumed almost double with 12.4 mL and both reached a pH 9.1–9.2. One possible explanation could be that under 100% H_2_ atmosphere more H_2_ is dissolved in the media. H_2_ has a low solubility in water (0.0014 g gas per kg water at 37 °C) and even lower in saline brine. So, growth will be limited by H_2_ dissolution, which will be increased with higher H_2_ concentration or pressure. Given the optimal growing conditions in the beginning (optimal pH and nutrients) and a high initial cell number, 100% H_2_ gives the cells more electron donor to be active in the initial growth phase compared to 40% H_2_. Also, concurrent chemical reactions of the H_2_ gas with the slightly alkaline media, leading to additional abiotic H_2_ loss, could be factor. Another reason could be that the increasing alkaline stress will trigger expression of pH-homeostasis related genes in *D.retbaense* cells. For *Desulfovibrio vulgaris* it was shown that several genes for ATP synthase, Na^+^/H^+^ antiporters and amino acid metabolism were expressed after an alkaline shock, which is a strategy to retain the cellular redox state and avoid an inverse pH gradient over the cell membranes^39,40^. These stress adaptations might also play a role in our tested strain and higher H_2_ concentrations might trigger a stronger or more complex gene expression, keeping the pH more stable. Further careful investigations combined with chemical and biological kinetic modelling and even including transcriptomic studies will help to understand the H_2_- and proton consumption.

The H_2_ electron chain towards sulphate is well understood and relatively conserved in members of the Deltaproteobacteria^41^, therefore it can be assumed that the observed effects of pH increase when grown on H_2_ is a general phenomenon and not strain specific.

Similarly, methanogenesis is a proton consuming process (reaction rate 2). Also, during methanogenesis the Archaea are consuming bicarbonate/CO_2_ from the media, which reduces buffer potential and increases pH as described previously^42^. We observed in our set-ups with *M. halotolerans* a pH increase, although not as intense as with *D. retbaense*. All H_2_ was consumed in all cases although activity rates decreased during the end of incubation for the 90% H_2_ bottles probably because pH increased and was close to the upper pH limit of this strain. Although the absolute rates were highest with the highest amount of H_2_, the relative consumption rates (= loss in %) with 10% H_2_ were the fastest and renewed addition of 10% H_2_ gave an even increased rate which is related to either increased cell numbers or increased activity. If this effect of higher relative rates with lower H_2_ concentrations will have an effect on the storage site, needs to be evaluated in the future.

### Potential risks for souring of salt caverns

Halophilic archaea and bacteria have been isolated from pure halite crystals in salt mines^43^ and it is therefore expected that that also salt caverns will be habitat to a diverse set of microbes. Recent studies of salt cavern microbiology showed microbes in all studied caverns^10^. Also, our studied salt cavern brine was microbiologically active and after several months of incubation with and without nutrient addition we observed H_2_ consumption. With addition of the growth booster yeast extract (supplying microbes with a variety of vitamins and trace elements) and the carbon source acetate, a significant H_2_ consumption was measurable together with acetate consumption, H_2_S production and also a significant pH increase. At the end of the incubation the pH increased 1 log factor from an initial 7.5 to 8.5. This shows that H_2_ oxidation by halophilic SRMs does indeed lead to a significant pH increase similarly to what we observed with our type strain *D. retbaense*. In the pure brine bottles without addition of carbon source or yeast extract, H_2_ consumption was less pronounced but still the presence of black iron sulfide minerals and a pH increase suggest that sulphate-reduction was on-going. This shows that the natural community is limited by a certain growth factor, trace element, vitamin or similar. We hope to be able to identify and enrich the sulphate-reducing community in future studies to better understand the growth behavior.

If we assume that the studied sulphate reducers will also be active in the salt cavern when in contact with H_2_, there is indeed a real risk for H_2_ loss and souring. The sampled cavern brine has a very high sulphate content of over 4000 mg/L (~ 44 mM), which gives sulphate-reducing organisms sufficient electron acceptors. A back of the envelope calculation of a typical salt cavern with a volume of 600.000 cubic meters (most volume will be occupied with gas but roughly 3000 m^3^ will be brine in the sump together with insoluble minerals) contains 12.6 metric tons of sulphate in the cavern to be potentially converted by sulphate-reducing organisms. However, in case of 100% sulphate conversion without any additional input and no inhibition/limitation, the cavern could contain around 3E+06 L liters of H_2_S or only 0.6% of the total cavern volume. Based on the equilibrium (1), four times the amount of H_2_ would be needed as electron source (1.2E+07 L), a loss of around 2% of the total volume. These values will depend on many factors including the physical cavern properties, the brine chemistry and our observed limiting effects of nutrients and pH. The consumption rates will firstly be dependent on the surface area of the brine-gas interphase and therefore the cavern shape. A higher surface area will allow for a higher H_2_ availability for the microbes. An important limiting factor will be the amount of available carbon source and/or growth factors, which led to a H_2_ loss of maximum 5–13% under laboratory conditions. Total inorganic carbon has been detected in the cavern brine of around 85 mg/L and some organic carbon of around 7.7 mg/L which was most likely introduced by the leaching process using diesel oil as a leaching blanket (personal communication with the cavern operators). This method of using diesel oil during leaching is/was very common and some organic carbon can be expected in most if not all salt-caverns. Other sources of organic carbon might also include the originally used leaching water (often sea water) or the presence of small amounts of organic compounds in the salt rock. Still, additional growth enhancers were necessary for significant sulphate reduction to occur, which slowed down when pH approached 9. This means that during the first H_2_ storage cycles, some volumes of H_2_ could be converted by microbes until the pH value will be outside of the optimum for these microbes and we speculate that the overall %-loss of H_2_ in a salt cavern due to microbial activity will be relatively low. As a cautionary note: the derived laboratory enrichments can only give hints about the metabolic potential in extreme environments since most microbes do not grow under lab conditions. A much more complex and chemolithoautotrophic community with higher cell numbers might be present and active in the cavern leading to a) faster H_2_ consumption and b) longer and more intense H_2_ consumption. For example, it could be that alkali-tolerant strains will take over H_2_ oxidation as soon as neutrophilic strains reached their limit or a certain adaptation to higher pH will occur. Indeed, given the production of methane after 170 days when pH was seemingly too high for the sulphate-reducing community, methanogenesis is inferred. Until the end of the experiment, we were not able to stimulate methanogenesis or acetogenesis directly by adding CO_2_ in our lab enrichments. As the brine seems to be very sensitive to pH changes due to low buffering material, addition of CO_2_ led to a strong pH decrease. However, the fact that we observed methane in our H_2_ + acetate + yeast shows that the microbial metabolism is possible and might cause a continued H_2_-loss after sulphate reduction. Based on our results with *M. halotolerans* it also seems that methanogens might be able to better cope with the pH increase, as the strain was able to consume all provided H_2_ even at pH > 8.5. This complex but slow interplay of the cavern community when in contact with H_2_ needs to be further studied.

Our data shows that artificially leached salt cavern can contain H_2_-consuming microbes, especially sulphate-reducers which seem to be nutrient limited and additionally will significantly increase pH. Field tests are now required to understand if the observed effects regarding H_2_ consumption and pH will also occur in the cavern itself.

## Conclusions

- Halophilic hydrogen-consuming sulphate reducers and methanogens are able to consume significant volumes of H_2_ over time.
- H_2_ oxidation leads to a significant pH increase in both cases. In case of the tested pure strain SRM, pH exceeds growth limits and H_2_ is not completely consumed.
- Original salt cavern brine contains active SRMs which are able to consume parts of the available H_2_ especially when carbon and nutrients were added. H_2_S gas was measurable in the headspace but decreased again when brine pH increased. Some produced H_2_S also precipitated with trace amounts of iron as iron-sulfide minerals
- The pH increase caused by H_2_ oxidizing-SRMs might limit microbial H_2_ consumption over long-term and might therefore be a self-limiting process in low-buffered environments like salt cavern brines.

## Supplementary Information

Supplementary Information.

## Data Availability

All main data generated or analysed during this study are included in this published article (and its Supplementary Information files).
