# Supplementary material for: Microbial hydrogen consumption leads to a significant pH increase under high-saline-conditions: implications for hydrogen storage in salt caverns
Source: Sci Rep. 2023 Jun 29;13:10564. doi: 10.1038/s41598-023-37630-y (PMC10310820; doi:10.1038/s41598-023-37630-y)
Supplement: Supplementary file 1 — Supplementary Information. [file 41598_2023_37630_MOESM1_ESM.pdf]

Turning basic on H_2: Microbial hydrogen consumption leads to intense pH increase under high-saline-conditions– implications for hydrogen storage in salt caverns

Nicole Dopffel[1], Kyle Mayers[1], Abduljelil Kedir[1], Edin Alagic [1], Daniel Boldt[2], Janiche Beeder[2], Silvan Hoth[2]*

SUPPLEMENTAL INFORMATION

Supplemental table 1: Experimental conditions for the hydrogen consumption experiments.

Strain used	T [°C]	H_2 [%]	CO_2 [%]	Yeast extract [%]	Acetate [mM]	Other additions	Inoculum [%]	Incubation [days]
D. retbaense	37	0	-	-	24	1.4 % vitamin solution	10	71
D. retbaense	37	10	-	-	24	1.4 % vitamin solution	10	71
D. retbaense	37	40	-	-	24	1.4 % vitamin solution	10	71
D. retbaense	37	100	-	-	24	1.4 % vitamin solution	10	71
Control	37	10	-	-	24	1.4 % vitamin solution	-	71
Control	37	40	-	-	24	1.4 % vitamin solution	-	71
Control	37	100	-	-	24	1.4 % vitamin solution	-	71
M. calculus	37	0	5	0.05	20	35 mM formate	10	9
M. calculus	37	10	5	0.05	20	35 mM formate	10	9
M. calculus	37	40	5	0.05	20	35 mM formate	10	9
M. calculus	37	90	5	0.05	20	35 mM formate	10	9
Control	37	10	5	0.05	20	35 mM formate	-	9
Control	37	40	5	0.05	20	35 mM formate	-	9
Control	37	90	5	0.05	20	35 mM formate	-	9
Cavern brine	30	100	-	-	-	-	-	176
Cavern brine	30	100	-	-	-	-	-	176
Cavern brine	30	100	-	0.04	20	-	-	176
Cavern brine	30	100	-	0.04	20	-	-	176
Cavern brine	30	90	10	-	-	-	-	176
Cavern brine	30	90	10	-	-	-	-	176
H_2O	30	100	-	-	-	-	-	211

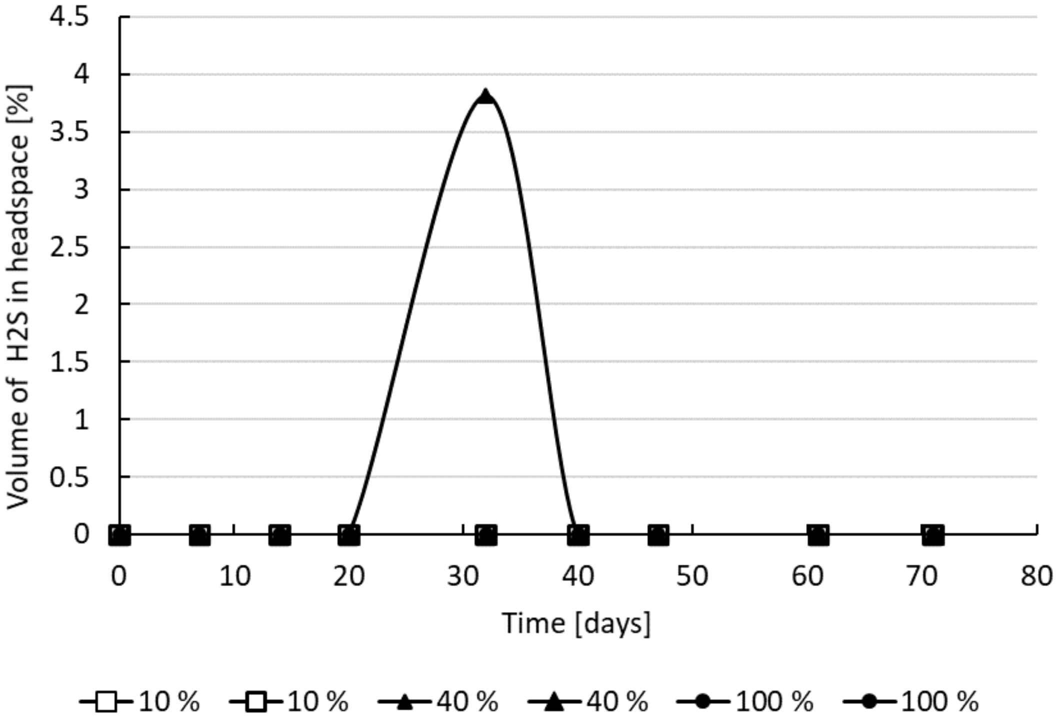

Suppl.Figure 1: produced H2S of *Desulfohalobium retbaense* in % over time measured in the headspace of bottles incubated at near atmospheric pressure and 37°C. Bottles were amended with 100% hydrogen (●, solid line), 40% hydrogen (▲, solid line) and 10% hydrogen (□, solid line). All individual bottles are shown. The values given in the table are minus the background level of H2S coming from the sodium sulfide in the media.

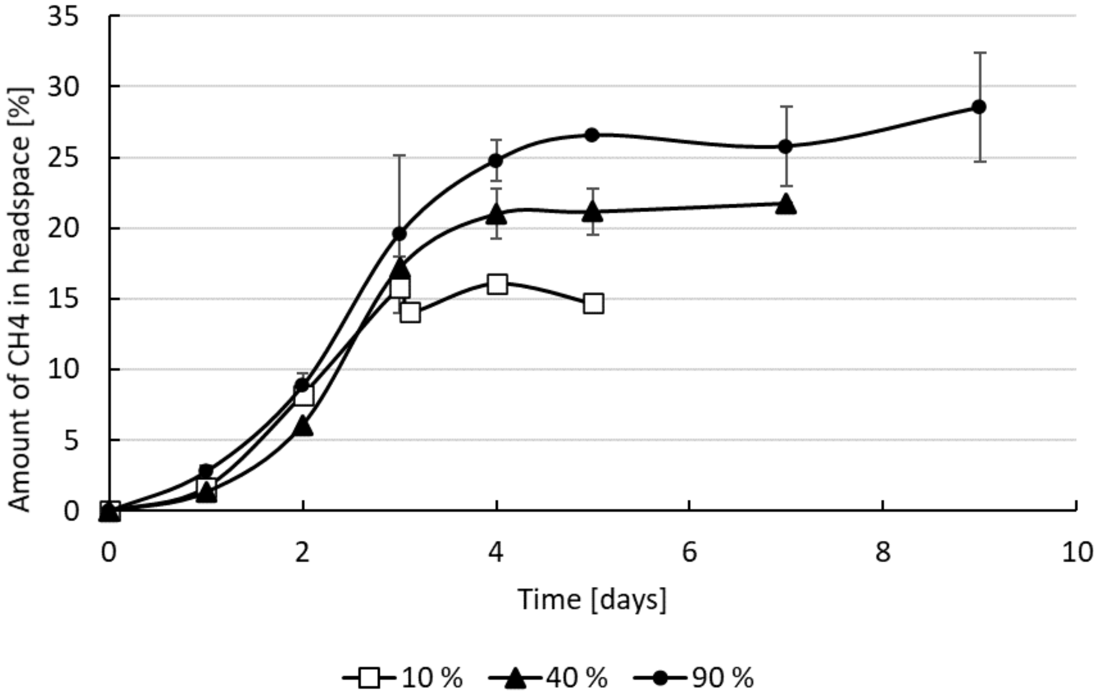

Suppl.Figure 2: Produced methane CH4 of *Methanocalculus halotolerans* in % of headspace over time of bottles incubated at near atmospheric pressure and 37°C. Bottles were amended with 90% hydrogen (●, solid line), 40% hydrogen (▲, solid line) and 10% hydrogen (□, solid line). Error bars indicate deviation from the mean of duplicates.

Supplemental table 2: Measured hydrogen amounts in mL in headspace over time given in days for all bottles and two independent experiments. Bottles started with 10, 40 or 100 or 90% hydrogen in the headspace.

D. retbaense

	Days														
% H2	**0**	**7**	**14**	**16**	**20**	**23**	**26**	**29**	**32**	**35**	**37**	**40**	**47**	**61**	**71**
**10**	3.9	1.7	0.9		0.5				0.0	3.4		2.9	2.5	1.8	1.5
**10**	4.0	1.9	1.1		0.7				0.1	3.6		3.1	2.8	2.1	1.8
**10**	3.8	1.4		0.3		0.1	2.6	3.3			2.9				
**10**	4.2	1.2		0.3		0.1	2.8	3.7			3.2				
**40**	12.2	8.7	7.8		6.9				4.9			3.8	3.7	3.7	3.7
**40**	10.8	8.2	7.6		6.6				5.3			4.4	4.2	4.1	4.2
**40**	13.9	8.8	6.5			5.4		4.8		4.2					
**40**	11.0	7.5	6.3			5.4		5.1		4.9					
**100**	25.0	19.6	16.0		13.4				10.8			8.8	8.6	8.3	8.3
**100**	24.8	21.5	19.5		16.7				14.2			12.7	12.8	12.9	12.9
**100**	33.7	26.8		23.1		20.9		16.4			16.4				
**100**	30.0	26.6		26.6		24.9		18.5			18.7				

M. halotolerans

	Days								
% H2	**0**	**1**	**2**	**3**	**3.1**	**4**	**5**	**7**	**9**
**10**	3.5	3.1	2.4	0.8	4.6	2.1	0.0	0.0	0.0
**10**	4.0	3.2	2.4	0.0	3.6	1.4	0.0	0.0	0.0
**10**	4.0	3.9	2.5	1.8					
**10**	3.5	3.7	2.4	1.7					
**40**	12.9	11.4	9.9	7.6		4.3	0.0	0.0	0.0
**40**	11.0	10.1	8.7	6.6		4.9	1.0	0.0	0.0
**40**	8.8	8.4	9.2	8.3			4.6	0.7	
**40**	8.8	8.5	9.6	8.8			5.8	2.4	
**90**	22.2	20.2	15.8	12.5		7.3	5.7	3.2	1.5
**90**	25.0	24.8	21.7	16.3		12.1	7.6	1.8	0.0
**90**	17.7	18.5	20.6	16.5			10.9	2.9	1.3
**90**	18.0	20.2	22.6	19.6			13.8	7.9	5.5

Supplemental table 3: Measured pH in the liquid phase over time given in days for all bottles. Bottles started with 10, 40 or 100 or 90% hydrogen in the headspace.

D. retbaense

	Days					
pH	**0**	**14**	**26**	**37**	**40**	**71**
**10**	7.6				8.5	8.9
**10**	7.6				8.5	8.9
**10**	7.6	7.5	8.5	8.8		
**10**	7.6	8.0	8.5	8.6		
**40**	7.6				8.5	9.1
**40**	7.6				9.0	9.2
**40**	7.6	9.0		9.0		
**40**	7.6	8.5		9.0		
**100**	7.6				9.0	9.1
**100**	7.6				9.0	9.1
**100**	7.6	8.5	8.5	9.1		
**100**	7.6	9.0	9.0	9.0		

M. halotolerans

	Days			
pH	**0**	**2**	**5**	**9**
**10**	7.5	8.3	8.6	
**10**	7.5	8.4	8.5	
**40**	7.5	8.1	8.5	
**40**	7.5	7.9	8.4	
**90**	7.4	7.9	8.6	8.8
**90**	7.4	7.9	8.7	8.7

Supplemental table 4: Starting and end values of a *D. retbaense* culture growing on 24 mM lactate at 37°C.

	Day 0	Day 7
pH	7.5	7.8
H_2S concentration in headspace [%]	1.1	14 *

* Please note that this H_2S concentration was far beyond our calibration in the microGC and therefore the value cannot be given with high confidence.
